# PbS Nanoparticles Prepared Using 1, 10-Phenanthroline Adduct of Lead(II) Bis(*N*-alkyl-*N*-phenyl dithiocarbamate) as Single Source Precursors

**DOI:** 10.3390/molecules25092097

**Published:** 2020-04-30

**Authors:** Jerry O. Adeyemi, Damian C. Onwudiwe

**Affiliations:** 1 Material Science Innovation and Modelling (MaSIM) Research Focus Area, Faculty of Natural and Agricultural Science, North-West University, Mafikeng Campus, Private Bag X2046, Mmabatho 2735, South Africa; jerryadeyemi1st@gmail.com; 2 Department of Chemistry, Faculty of Natural and Agricultural Science, North-West University, Mafikeng Campus, Private Bag X2046, Mmabatho 2735, South Africa

**Keywords:** dithiocarbamate, adducts, precursor, solvothermal, nanoparticles, morphology

## Abstract

Dithiocarbamate complexes have remained prominent as single source precursors for the synthesis of clean metal sulfide nanoparticles. This study reports the synthesis of lead sulfide (PbS) nanoparticles using some novel complexes of 1, 10-phenanthroline lead(II) bis(*N*-alkyl-*N*-phenyl dithiocarbamate), represented as [Pb(L^1^)_2_phen] (1) and [Pb(L^2^)_2_phen] (2) (where L^1^ = bis(*N*-ethyl-*N*-phenyldithiocarbamate; L^2^ = bis(*N*-butyl-*N*-phenyldithiocarbamate); phen = 1, 10 phenanthroline) as a single source precursors. The complexes (1 and 2) were synthesized and characterized using various spectroscopic techniques and elemental analysis. The nanoparticles were synthesized via a solvothermal approach in oleylamine, used as a capping agent, and were given as PbS(1) and PbS(2) from [Pb(L^1^)_2_phen] (1) and [Pb(L^2^)_2_phen] (2), respectively, which were characterized using X-ray diffraction (XRD), transmission electron microscopy (TEM), and absorption spectroscopy. The diffraction patterns confirmed the formation of face-centered cubic phase PbS nanoparticles with a preferential growth orientation along the (200) plane. The TEM images showed that PbS(1) were of a spherical morphology, while the morphology of PbS(2) tended to produce short rods. This was due to variation in the functional group on the precursor compounds. This variation also resulted in the different band gap energies found such as 1.148 and 1.107 eV for PbS(1) and PbS(2), respectively, indicating a blue shift from the bulk.

## 1. Introduction

Lead sulfide (PbS) belongs to an important class of semiconductors (IV-VI group) and possesses a narrow band gap of 0.41 eV, with an exciton Bohr radius of 18 nm at room temperature [1,2]. It also possesses a high dielectric constant and carrier mobility, which consequently produces a strong quantum size effect regardless of the often observed large sizes [2]; thus, making it an ideal material for studying quantum size effects [1]. Many approaches for the synthesis of PbS nanoparticles have stemmed from the increased interest in PbS materials, due to their useful physical properties and application in many optoelectronic devices [1]. The synthetic approach to making this material with the desired properties, size and shape are of utmost importance. These routes could differ in parameters such as the nature of precursor, reaction conditions and techniques used [3]. One such useful synthetic approach is the solvothermal decomposition of a single source precursor, in the presence of a surfactant such as oleylamine [1].

The solvothermal method of synthesis has continued to gain attention in the synthesis of metal chalcogenide nanoparticles [4]. It is a technique involving the decomposition of a single source precursor in a solvent whose high boiling point is beyond the stability of the precursor compound. Dithiocarbamate complexes are widely used single source precursors, as they have been reported to undergo clean thermal decomposition to produce metal sulfide nanoparticles with little or no impurity. This property is due to the exceptional binding ability of the dithiocarbamato anion, which forms a metal-sulfur bond with most transition metals [5]. This binding ability has been found to arise from the presence of two-electron donor sulfur atoms, which in turn aids the stability of the complex [6]. Furthermore, a notable advantage of this single source precursor is the ability to fine tune the properties of the synthesized nanoparticle by tweaking the dithiocarbamate backbone to influence features such as shape, size and optical properties of the desired material [7]. The O’Brien group was the first to report the use of single source precursor of lead(II) dithiocarbamate complex [8]. Lead(II) complexes derived from piperidine and tetrahydroquinoline dithiocarbamate ligands have also been used as single source precursors by Nyamen et al. for PbS nanoparticles using hexadecylamine, dodecylamine, decylamine and oleylamine as capping agents to produce particles of varying shapes [9]. Onwudiwe has also reported the microwave-assisted synthesis of PbS using complexes of lead(II) bis(*N*-alkyl- *N*-phenyldithiocarbamate), which produced varying shapes such as nanorods and nanocubes [10]. In continuation of this study, we herein investigate the solvothermal synthesis of PbS, using 1, 10 phenanthroline adducts of lead(II) bis(*N*-alkyl-*N*-phenyldithiocarbamate) as single source precursors and oleylamine as a capping agent.

## 2. Results and Discussion

### 2.1. Synthesis

**Scheme 1:**
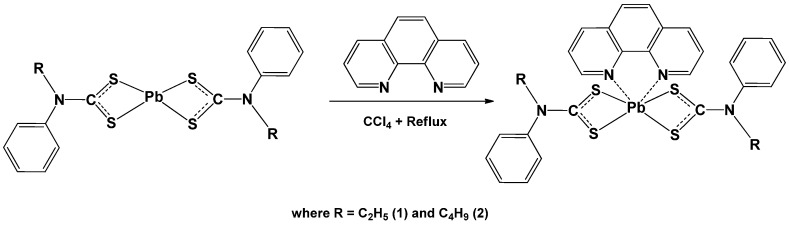
Reaction scheme for the preparation of 1, 10 phenanthroline lead(II) *N*-alkyl-*N*-phenlydithiocarbamate complexes.

**Scheme 2:**
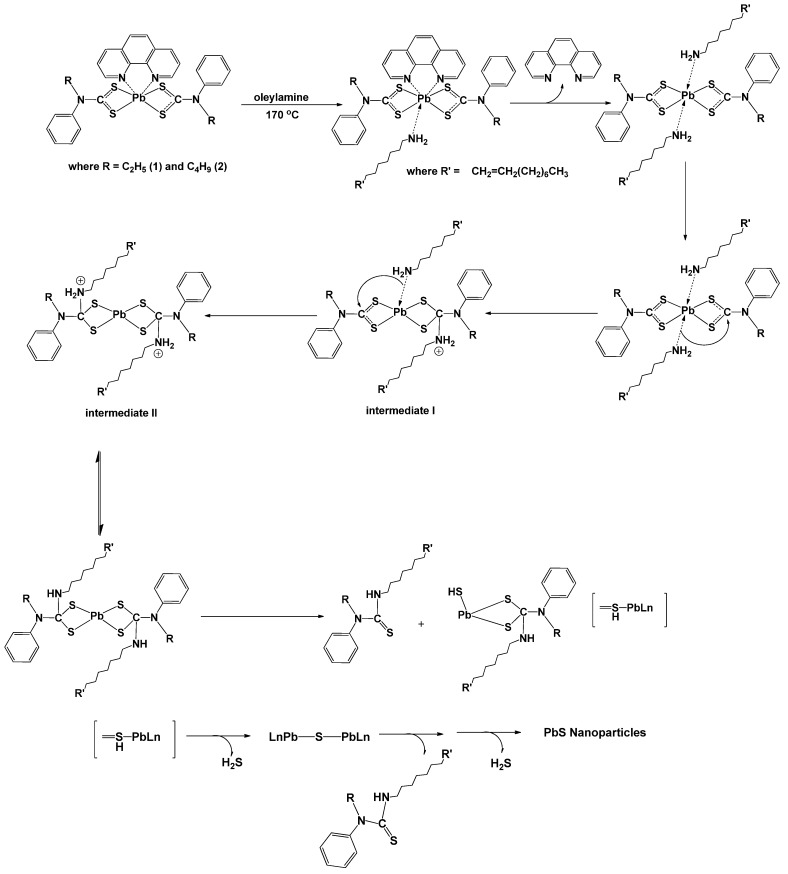
Proposed schematic pathway for the thermal decomposition of 1, 10 phenanthroline lead(II) *N*-alkyl-*N*-phenlydithiocarbamate complexes in the presence of oleylamine.

The adducts were prepared by refluxing the parent complexes of lead(II) bis(*N*-ethyl-*N*-phenyl dithiocarbamate) and lead(II) bis(*N*-butyl-*N-*phenyldithiocarbamate) [10], in chloroform in the presence of 1,10-phenanthroline as shown in Scheme 1. In this reaction, the 1,10-phenanthroline acted as a Lewis base and donated its lone pair electrons to the d-orbital of Pb atom in the precursor complexes to form adducts. These adducts were very stable at room temperature, and were generally soluble in dichloromethane, tetrahydrofuran and dimethylsulfoxide. The resulting adduct was solvo-thermally decomposed in oleylamine to produce PbS nanoparticles. A proposed schematic pathway is presented in Scheme 2, similar to the previously proposed pathway in a comparable study involving dithiocarbamate compound [11].

### 2.2. Spectroscopic Study

In the infrared (IR) spectra of both precursor complexes provided in Figure S1, the stretching frequencies of the (C-N) bonds was observed to decrease to lower frequencies of 1350 and 1352 cm^−1^ [10] for complexes **1** and **2**, respectively. This was a decrease from the (C-N) bond of the parent complexes observed at ≈ 1470 [10]. This may be due to a change in the coordination number at the metal center, and a possible steric effect exerted by the introduction of 1,10-phenanthroline base [12]. Furthermore, the ring frequencies of the 1,10-phenanthroline, which are usually found in the frequency range of 1600–1000 cm^−1^ [13], were mostly masked by bands due to the dithiocarbamate ligand which also exists within the same range. The presence of a single stretching band of (C=S), although in a higher range when compared to the parent complexes, showed that the dithiocarbamate ligand remained bonded in a bidentate fashion to the Pb(II) atom in the adducts.

In the ^1^H NMR spectra obtained for both complexes (Figures S2 and S3), multiplets were found in the downfield region between 9.20 and 7.47 ppm which are assigned to the aromatic ring of the 1, 10 phenanthroline moiety [14]. Similarly, multiplets originating from the aromatic ring of the dithiocarbamate moiety were found in the region between 7.45 and 6.59 ppm in both complexes, partly overlapping with the signals of the 1, 10 phenanthroline moiety. Furthermore, methylene and methyl protons from both complexes were found in the region between 4.26 and 0.86 ppm, in each case similar to other reports involving the use of the same ligands [15,16]. In the ^13^C NMR spectra, the most important chemical shift of the thioureide carbon bond was found for both complexes at an approximate position of 210 ppm, which is further downfield compared to the parent complexes of **1** and **2** [10]. This is due to additional dishielding effect caused by the presence of 1, 10 phenanthroline moiety [14]. This additional bonding of the nitrogenous base to the lead dithiocarbamate center produced a decrease in the partial double bond character of the N꞊C bond; consequently resulting in the displacement of electron density from carbon to nitrogen of the dithiocarbamate ligand [14,17]. Other chemical shifts which supported the existence of the adduct were found in the region between 149 and 121 ppm and have been assigned to the aromatic carbons of the 1, 10 phenanthroline group.

### 2.3. The X-ray Diffraction Study of the Synthesized PbS Nanoparticles

**Figure 1:**
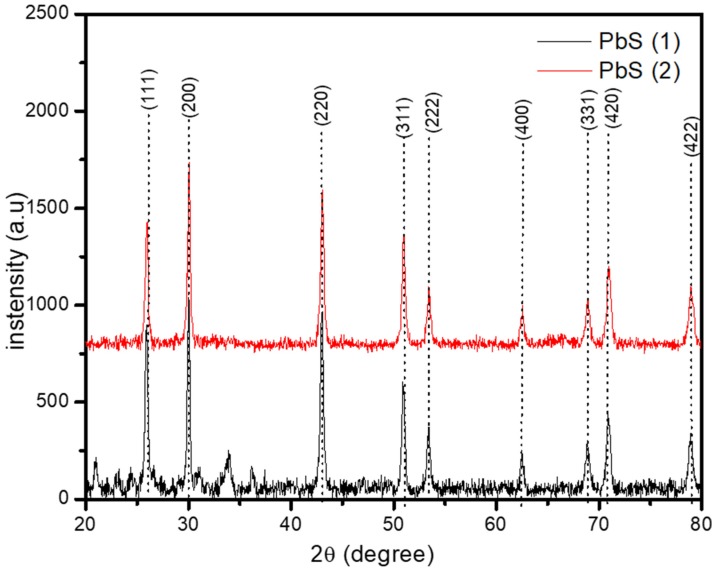
X-ray Diffraction (XRD ) patterns of PbS synthesized from 1, 10 phenanthroline lead(II) bis(*N*-alkyl-*N*-phennyl dithiocarbamate) adducts.

The crystalline phase and chemical composition of the PbS nanoparticles (NPs) have been obtained by XRD, in the range 20° to 80° as presented in Figure 1. The diffraction peaks at 2θ values of 26°, 30°, 43°, 51°, 53°, 63°, 69°, 71° and 79°, have been indexed to the (1 1 1), (2 0 0), (2 2 0), (3 1 1), (2 2 2), (4 0 0), (3 3 1), (4 2 0) and (4 2 2) peaks of the face-centred cubic (fcc) phase of PbS (galena) with a lattice constant of a = 5.93620 Å (according to Joint Committee on Powder Diffraction (JCPD) No: 005-0592). This confirms the formation of PbS nanoparticles. The sharp narrow peaks observed are indicative of the good crystallinity of the NPs [18]. The ratio of the intensity of the (111) to the (200) diffraction peaks were compared with 0.976 of the standard sample. The smaller values of 0.8519 and 0.6784 for PbS(1) and PbS(2), respectively, compared to the standard sample indicated a preferred orientation along the (200) plane, similar to previous reports [3,19]. This also showed that the growth rate on the (111) facet was much lower than the (100) facet [20]. These results showed that, although PbS(1) and PbS(2) were synthesized under similar conditions, they exhibited different peak intensity but achieved the same pattern regardless of the geometrical differences in the precursor compounds. Other preferential orientation has been observed as the temperature increases for PbS nanoparticles, with the (220) preferred growth orientation at 200 °C [3].

### 2.4. Morphology of the Synthesized PbS Nanoparticles

**Figure 2:**
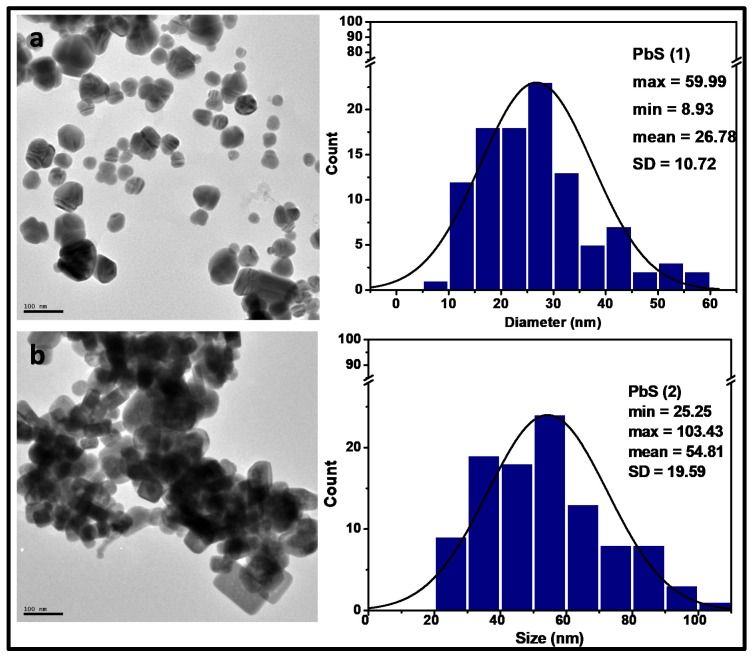
Transmission electron microscopy (TEM) micrographs showing the morphology of synthesized (**a**) PbS(1) and (**b**) PbS(2) nanoparticles with their respective particle size distribution histogram. SD stands for standard deviation.

The transmission electron microscopy (TEM) images and the particle-size distribution histogram, which shows the size range for the width of particles for a count of over 100 particles, have been obtained and presented in Figure 2. The morphology of the nanoparticles showed reasonably dispersed spherical shaped particles for **PbS(1)**, while those observed for **PbS(2)** exhibited some anisiotropic morphology in the form of short rods with slight agglomeration. These obtained morphologies are comparable to those reported in literature at a similar temperature range of preparation [3]. The estimated average particle diameters were found to be 26.78 ± 10.72 and 54.813 ± 19.59 nm for **PbS(1)** and **PbS(2)**, respectively. These particle diameters were smaller than those reported for a similar study involving the use of lead ethyl xanthogenate as a single source precursor to PbS nanoparticles [3]. Thus, the effect of the use of different precursors was obvious in the shape and sizes of the as-synthesized PbS nanoparticles. The **PbS (1)** derived from complex 1, bearing the shorter chain (ethyl group) in the dithiocarbamate backbone, produced more uniform, spherical shaped particles, while those of **PbS (2)** bearing the longer butyl derivatives resulted in the formation of short rods. Furthermore, the observed narrow peaks in the obtained XRD plot agree with the estimated average size of the particle obtained in the TEM images, indicating a large size due to the quick depletion of the precursors at elevated temperature, prompting the Ostwald ripening process, in which larger particles grow at the expense of smaller particles [3,10]. Varied morphologies have been reported for PbS nanoparticles derived from single source precursors and this is predominantly influenced by the temperature of thermolysis and the precursor used [21]. Akhtar et al. [1] reported the thermolysis of some derivatives of dialkyldithiocarbamato lead(II) in oleylamine at 60 °C and 80 °C, which resulted in the formation of almost spherical and cubic PbS nanoparticles with the same precursor. Other derivatives of this group of complexes did not produce the expected nanoparticles at these temperatures. However, at 150 °C, these complexes also produced spherical morphologies within 30 min of thermal decomposition [1].

### 2.5. UV-Vis-Near Infrared Spectral Study (Optical Properties)

**Figure 3:**
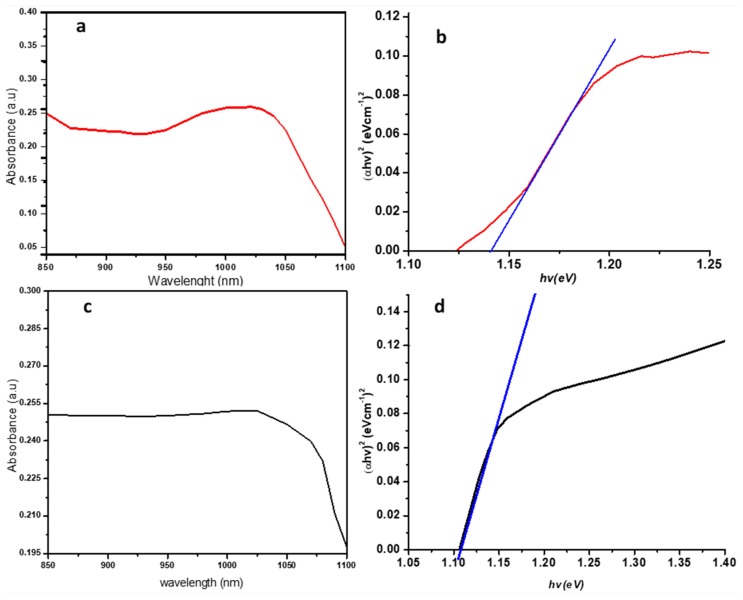
UV-Vis-NIR (Ultra Violet-Visible-Near Infrared) absorption spectrum (**a**,**c**) and Tauc (**b**,**d**) plot of the synthesized PbS nanoparticle (1) red and (2) black.

UV–visible-near infrared absorption spectroscopy has been employed to study the optical properties of the synthesized nanoparticles. At maximum exciton absorption, it is generally known that the wavelength decreases as the size of the nanoparticles decrease, due to quantum confinements of the photogenerated electron-hole carriers [22]. As observed in Figure 3a,c, the excitonic peak was observed for PbS(1) and PbS(2) at 1030 and 1025 nm, respectively. Lead sulfide is a direct band gap semiconductor and thus a plot of (αhʋ)^2^ against hʋ showed a linear portion that corresponded to the energy of the optical band gap when extrapolated to the hʋ axis [6]. Thus, Tauc’s plot was used to estimate the optical properties that produced the band gaps for both PbS(1) and PbS(2) at 1.148 and 1.107 eV, respectively, as shown in Figure 3b,d. These observed band gaps were blue-shifted away from the bulk, whose value is 0.41 eV, thus, conformed to other literature reports [6,10].

## 3. Materials and Methods

All chemicals were obtained from Merck chemicals (Darmstadt, Germany) and used without further purification. The ^1^H and ^13^C-NMR measurements in dimethyl sulfoxide (DMSO), were obtained using a Bruker Avance III 600 MHz (Billerica, MA, USA). Elemental C, H, N, and S (in %) analyses were obtained using an Elementar Vario EL Cube (Langenselbold, Germany), while the infrared spectra were recorded on a Bruker Alpha-P FTIR spectrophotometer. The crystalline phases of the synthesized materials were identified using X-ray diffraction (XRD) measurements at a scanning rate of 0.0018 °/min, using a Rőntgen PW3040/60 X’Pert Pro XRD (Shanghai, China) diffractometer which is equipped with a nickel filtered Cu Ka radiation (k = 1.5418 Å) at room temperature. The UV-Vis-NIR spectra were obtained from a Jobin Yvon LabRAM HR 800 UV-VIS-NIR (Horiba, Edison, NJ, USA) spectrophotometer in toluene. The morphology of the nanoparticles was characterized using a TECNAI G2 (ACI) TEM (Hillsboro, OR, USA) with an accelerating voltage of 200 kV.

### 3.1. Synthesis of 1, 10-Phenanthroline lead(II) bis(N-ethyl-N-phenyldithiocarbamate) [Pb(L^1^)_2_phen] (1)

10 mL of hot chloroform solution of 1, 10 phenanthroline (0.27 g, 1.50 mmol) was added to a hot 35 mL solution of bis(*N*-ethyl-*N*-phenyldithiocarbamate) (0.75 g, 1.25 mmol) in the same solvent. The resulting yellow solution was refluxed for 20 min, then concentrated to about 20 mL and filtered. The bright yellow solids which separated out from the solution, after 48 h, were filtered and dried over anhydrous calcium chloride.

[Pb(L^1^)_2_phen] (1): Yield: 91%; m.p.: 196–198 °C; Selected FTIR, υ (cm^−1^): 1437 (C=N), 1350 (C_2_-N), 1007 (C=S), 2973, 2925, 2864 (-CH), 3056 (=CH); ^1^H NMR (CDCl_3_) δ (ppm) = 9.07–7.47 (m, 8H, N_2_-C_12_H_8_), 7.40–7.16 (m, 10H, N-C_6_H_5_), 4.12 (q, 4H, N–CH_2_), 1.24 (t, 6H-CH_3_); ^13^C NMR (DMSO) δ (ppm) = 209.7 (-NCS_2_), 149.1–121.0 (N_2_-C_12_H_8_), 140.2–125.1 (N-C_6_H_5_), 47.1 (N–CH_2_), 12.8 (-CH_3_)

C_30_H_28_N_4_PbS_4_: C, 46.19; H, 3.62; N, 7.18; S, 16.44. Found C, 46.22; H, 3.58; N, 7.11; S, 15.97%.

### 3.2. Synthesis of 1, 10-Phenanthroline lead(II) bis(N-butyl-N-phenyldithiocarbamate) [Pb(L^2^)_2_phen] (2)

The synthesis of [Pb(L^2^)_2_phen] (2) followed the same procedure as described for [Pb(L^1^)_2_phen] (1), except that 1, 10 phenanthroline bis(*N*-butyl-*N*-phenyldithiocarbamate) was used in place of 1, 10 phenanthroline bis(*N*-ethyl-*N*-phenyldithiocarbamate). The product was filtered, washed with ethanol-water, and dried under vacuum.

[Pb(L^2^)_2_phen] (2): Yield: 90%, m.p.: 161–162 °C.; Selected FTIR, υ (cm^−1^): 1488 (C=N), 1352 (C_2_-N), 1000 (C=S), 2971, 2925, 2855 (-CH), 3042 (=CH); ^1^H NMR (CDCl_3_) δ (ppm) = 9.62–7.72 (m, 8H, N_2_-C_12_H_8_), 7.40–6.59 (m, 10H, N-C_6_H_5_), 4.26 (t, 4H, N–CH_2_CH_2_CH_2_CH_3_), 1.69 (m, 4H, N–CH_2_CH_2_CH_2_CH_3_), 1.21 (m, 4H, N–CH_2_CH_2_CH_2_CH_3_), 0.86 (t, 4H, N–CH_2_CH_2_CH_2_CH_3_); ^13^C NMR (DMSO) δ (ppm) = 209.5 (-NCS_2_), 149.1–121.0 (N_2_-C_12_H_8_), 140.0–125.7 (N-C_6_H_5_), 46.3 (N-CH_2_CH_2_CH_2_CH_3_), 29.6 (N-CH_2_CH_2_CH_2_CH_3_), 21.2 (N-CH_2_CH_2_CH_2_CH_3_), 12.6 (N-CH_2_CH_2_CH_2_CH_3_);

C_34_H_36_N_4_PbS_4_: C, 48.84; H, 4.34; N, 6.70; S, 15.34. Found C, 48.82; H, 4.21; N, 6.68; S, 15.30%

### 3.3. Synthesis of Lead Sulfide Nanoparticles

The adopted method of synthesis was similar to an already reported procedure with slight modification [23]. About 0.3 g of the Pb(II) complex (**1** or **2**) was dispersed in 10 mL of oleylamine in a 250 mL, three-necked round-bottom flask and stirred. The obtained mixture was degassed for 10 min at room temperature, followed by heating up to 120 °C and then held for 20 min under nitrogen. Afterwards, the temperature was increased to 170 °C and maintained for 1 h. After the reaction, the product was cooled to around 70 °C, followed by the addition of methanol for the precipitation of the nanoparticles. The product was then centrifuged and repeated washing and centrifuging produced pure nanoparticles.

## 4. Conclusions

New adducts of dithiocarbamate derived from 1, 10 phenanthroline and lead(II) bis(*N*-alkyl-*N*-phenyldithiocarbamate) complexes were synthesized and characterized. These complexes were used as a single source precursor for PbS nanoparticles via a solvothermal method. Both nanoparticles exhibited the same PbS phases, under similar synthetic conditions, and the same pattern was also obtained regardless of the compositional and geometrical differences in the used precursors. However, different intensities in peaks were observed. The obtained TEM micrographs showed spherical morphology in **PbS (1)** derived from a shorter ethyl chain group in the dithiocarbamate backbone of complex 1**,** while short rods with slight agglomeration were observed in **PbS(2),** involving a bulkier butyl group in the dithiocarbamate complex 2. Furthermore, both nanoparticles produced direct band gaps of 1.148 and 1.107 eV for both PbS(1) and PbS(2), respectively, indicating their ability to potentially harvest photons from the sunlight. This report suggests that, apart from functionalizing the backbone of a dithiocarbamate to encourage morphological changes, the utilization of adducts with variation in structures can also result in both structural and morphological changes in the properties of nanomaterials.

## Supplementary Materials

The following are available online, Figure S1: FTIR spectra of the Adduct Pb(II) complexes [Pb(L1)2phen] (A) and [Pb(L2)2phen] (B), Figure S2: ^1^H (A) and ^13^C NMR (B) spectra of the adduct complex [Pb(L1)2phen], Figure S3: ^1^H (A) and ^13^C NMR (B) spectra of the adduct complex [Pb(L2)2phen].

## References

[1] Akhtar J., Malik M.A., O’Brien P., Helliwell M. (2010). Controlled synthesis of PbS nanoparticles and the deposition of thin films by Aerosol-Assisted Chemical Vapour Deposition (AACVD). J. Mater. Chem..

[2] Himadri D., Pranayee D., Kumar S.K. (2018). Synthesis of PbS Nanoparticles and Its Potential as a Biosensor based on Memristic Properties. J. Nanosci. Technol..

[3] Tshemese Z., Khan M.D., Mlowe S., Revaprasadu N. (2018). Synthesis and characterization of PbS nanoparticles in an ionic liquid using single and dual source precursors. Mater. Sci. Eng. B.

[4] Tohidi T., Jamshidi-Ghaleh K., Namdar A., Abdi-Ghaleh R. (2014). Comparative studies on the structural, morphological, optical, and electrical properties of nanocrystalline PbS thin films grown by chemical bath deposition using two different bath compositions. Mater. Sci. Semicond. Process..

[5] Roffey A.R. (2014). Dithiocarbamate Complexes as Single Source Precursors to Metal Sulfide Nanoparticles for Applications in Catalysis. Ph.D. Thesis.

[6] Saah S.A., Boadi N.O., Adu-Poku D., Wilkins C. (2019). Lead ethyl dithiocarbamates: Efficient single-source precursors to PbS nanocubes. R. Soc. Open Sci..

[7] Ajibade P.A., Onwudiwe D.C., Moloto M.J. (2011). Synthesis of hexadecylamine capped nanoparticles using group 12 complexes of N-alkyl-N-phenyl dithiocarbamate as single-source precursors. Polyhedron.

[8] Trindade T., Brien P.O. (1997). Lead(II) dithiocarbamato complexes as precursors for the LP-MOCVD of lead sulfide. Chem. Vap. Depos..

[9] Nyamen L.D., Rajasekhar Pullabhotla V.S.R., Nejo A.A., Ndifon P.T., Warner J.H., Revaprasadu N. (2012). Synthesis of anisotropic PbS nanoparticles using heterocyclic dithiocarbamate complexes. Dalt. Trans..

[10] Onwudiwe D.C. (2019). Microwave-assisted synthesis of PbS nanostructures. Heliyon.

[11] Aamir A., Khan Y., Rehman M., Lin D. (2020). Catalytic and photocatalytic efficacy of hexagonal CuS nanoplates derived from copper (II) dithiocarbamate. Mater. Chem. Phys..

[12] Ajibade P.A., Onwudiwe D.C. (2013). Synthesis, characterization and thermal studies of 2,2′-bipyridine adduct of bis-(N-alkyl-N-phenyl dithiocarbamato-S,S′)cadmium(II). J. Mol. Struct..

[13] Srinivasan N., Thirumaran S., Ciattini S. (2014). Synthesis of α-mercury sulfide nanosheets from (1,10-phenanthroline)bis(1,2,3,4-tetrahydroquinolinecarbodithioato-S,S′)mercury(II). J. Mol. Struct..

[14] Sathiyaraj E., Padmavathy K., Kumar C.U., Krishnan K.G., Ramalingan C. (2017). Synthesis and spectral studies on Cd(II) dithiocarbamate complexes and their use as precursors for CdS nanoparticles. J. Mol. Struct..

[15] Kamaludin N.F., Awang N., Baba I., Hamid A., Meng C.K. (2013). Synthesis, characterization and crystal structure of organotin(IV) N-Butyl-N-phenyldithiocarbamate compounds and their cytotoxicity in human leukemia cell lines. Pakistan J. Biol. Sci..

[16] Adeyemi J.O., Onwudiwe D.C., Ekennia A.C., Okafor S.N., Hosten E.C. (2019). Organotin(IV) N-butyl-N-phenyldithiocarbamate complexes: Synthesis, characterization, biological evaluation and molecular docking studies. J. Mol. Struct..

[17] Rani P.J., Thirumaran S. (2013). Synthesis, characterization, cytotoxicity and antimicrobial studies on bis(*N*-furfuryl-*N*-(2-phenylethyl)dithiocarbamato-S,S′)zinc(II) and its nitrogen donor adducts. Eur. J. Med. Chem..

[18] Zhao Z., Zhang K., Zhang J., Yang K., He C., Dong F., Yang B. (2010). Synthesis of size and shape controlled PbS nanocrystals and their self-assembly. Colloids Surf. A Physicochem. Eng. Asp..

[19] Seghaier S., Kamoun N., Brini R., Amara A.B. (2006). Structural and optical properties of PbS thin films deposited by chemical bath deposition. Mater. Chem. Phys..

[20] Liu M., Li W. (2018). Growth and optical property of PbS/ZnS nanocrystals. Superlattices Microstruct..

[21] Mubiayi K.P., Revaprasadu N., Garje S.S., Moloto M.J. (2017). Designing the morphology of PbS nanoparticles through a single source precursor method. J. Saudi Chem. Soc..

[22] Sathiyaraj E., Thirumaran S. (2012). Synthesis and spectral studies on Pb(II) dithiocarbamate complexes containing benzyl and furfuryl groups and their use as precursors for PbS nanoparticles. Spectrochim. Acta-Part A Mol. Biomol. Spectrosc..

[23] Adeyemi J.O., Onwudiwe D.C., Hosten E.C. (2019). Synthesis, characterization and the use of organotin(IV) dithiocarbamate complexes as precursor to tin sulfide nanoparticles by heat up approach. J. Mol. Struct..

