# Supplementary material for: PbS Nanoparticles Prepared Using 1, 10-Phenanthroline Adduct of Lead(II) Bis(N-alkyl-N-phenyl dithiocarbamate) as Single Source Precursors
Source: Molecules. 2020 Apr 30;25(9):2097. doi: 10.3390/molecules25092097 (PMC7248792; doi:10.3390/molecules25092097)
Supplement: Supplementary file 1 [file molecules-25-02097-s001.pdf]

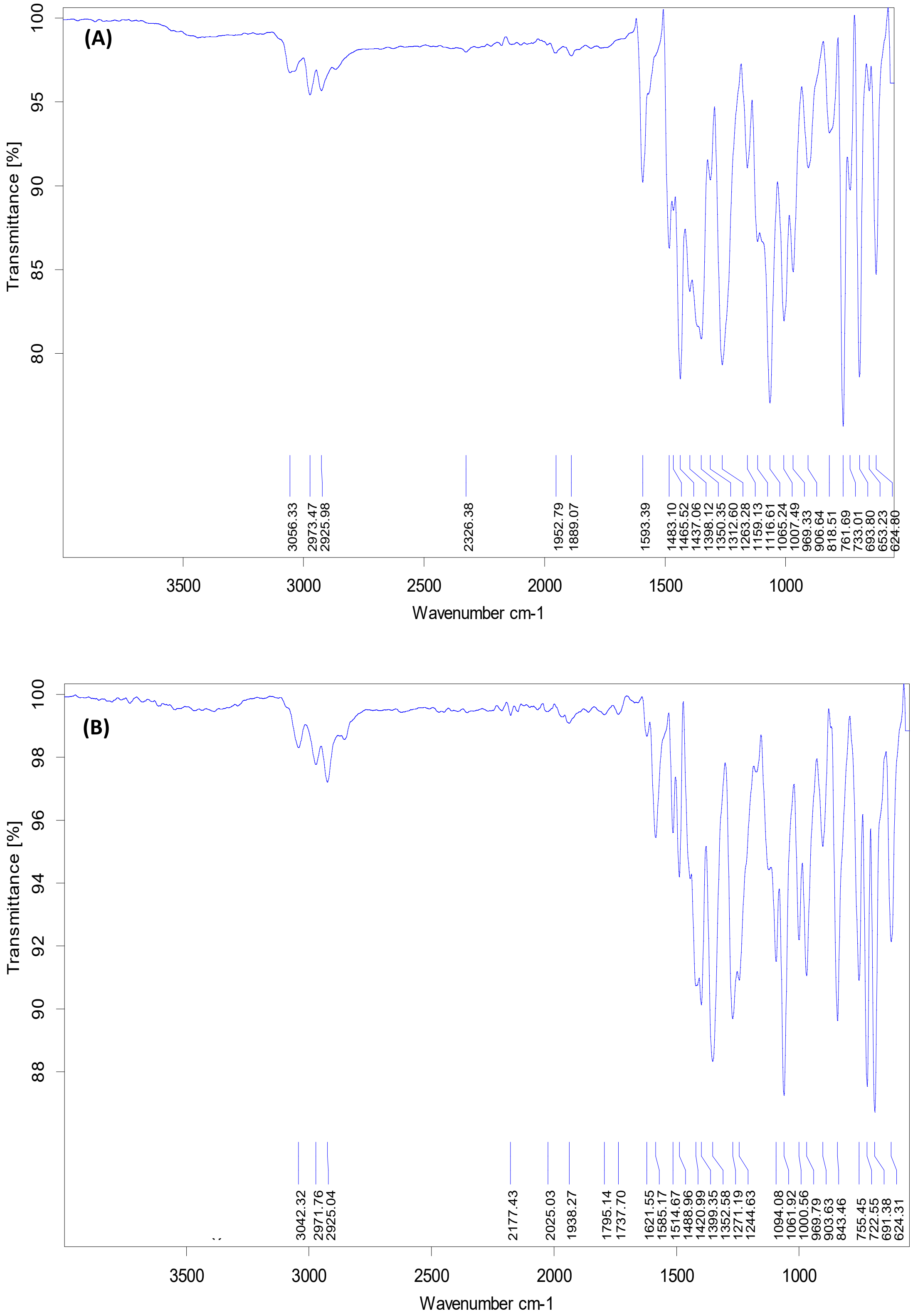

Figure S1. FTIR spectra of the Adduct Pb(II) complexes **[Pb(L[1])$_2$phen]** (A) and **[Pb(L[2])$_2$phen]** (B).

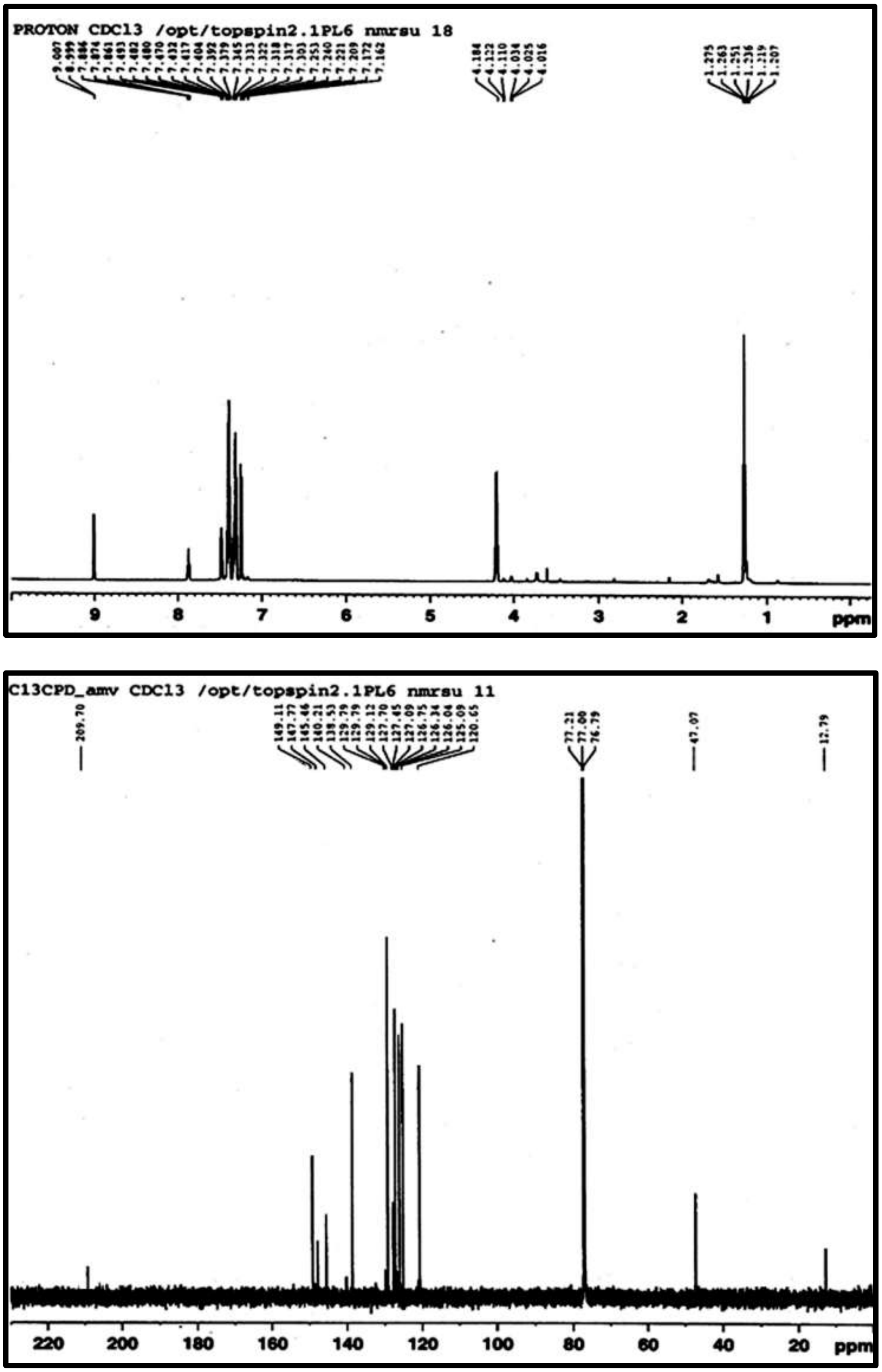

Figure S2. ^{1}H (**A**) and ^{13}C NMR (**B**) spectra of the adduct complex **[Pb(L^{1})$_2$phen].**

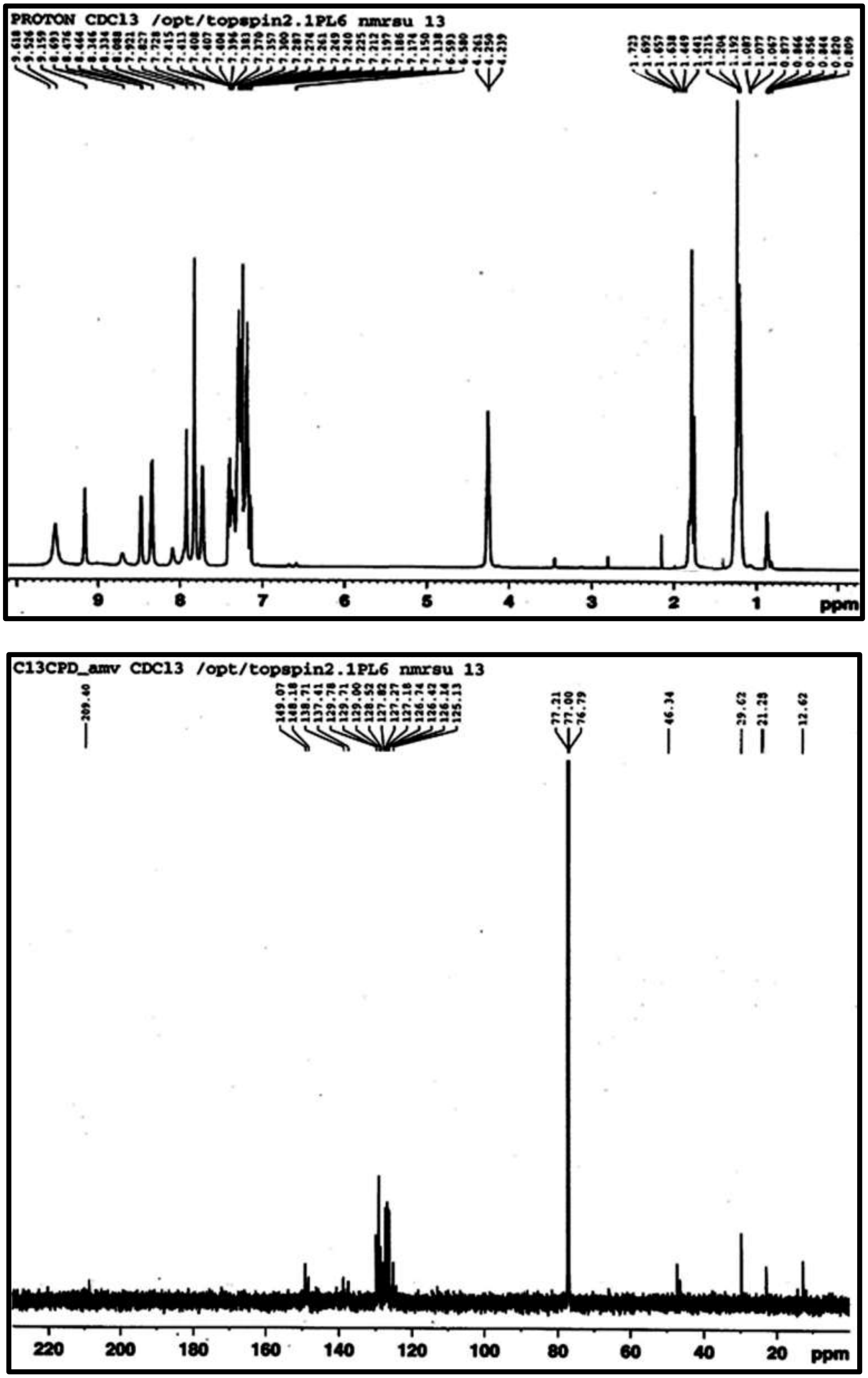

Figure S3. ^{1}H (**A**) and ^{13}C NMR (**B**) spectra of the adduct complex **[Pb(L^{2})$_{2}$phen].**
